# Resolution Enhancement Strategies in Photoacoustic Microscopy: A Comprehensive Review

**DOI:** 10.3390/mi15121463

**Published:** 2024-11-30

**Authors:** Jinying Zhang, Yifan Shi, Yexiaotong Zhang, Haoran Liu, Shihao Li, Linglu Liu

**Affiliations:** 1 Beijing Key Laboratory for Precision Optoelectronic Measurement Instrument and Technology, School of Optics and Photonics, Beijing Institute of Technology, Beijing 100081, China; syf188115@163.com (Y.S.); 3120220661@bit.edu.cn (Y.Z.); liuhaoran163@163.com (H.L.); lshao163@163.com (S.L.); lll12300901@163.com (L.L.); 2 Yangtze Delta Region Academy of Beijing Institute of Technology, Jiaxing 314001, China

**Keywords:** photoacoustic microscopy, acoustic-resolution photoacoustic microscopy, optical-resolution photoacoustic microscopy, resolution enhancement

## Abstract

Photoacoustic imaging has emerged as a promising modality for medical imaging since its introduction. Photoacoustic microscopy (PAM), which is based on the photoacoustic effect, combines the advantages of both optical and acoustic imaging modalities. PAM facilitates high-sensitivity, high-resolution, non-contact, and non-invasive imaging by employing optical absorption as its primary contrast mechanism. The ability of PAM to specifically image parameters such as blood oxygenation and melanin content makes it a valuable addition to the suite of modern biomedical imaging techniques. This review aims to provide a comprehensive overview of the diverse technical approaches and methods employed by researchers to enhance the resolution of photoacoustic microscopy. Firstly, the fundamental principles of the photoacoustic effect and photoacoustic imaging will be presented. Subsequently, resolution enhancement methods for both acoustic-resolution photoacoustic microscopy (AR-PAM) and optical-resolution photoacoustic microscopy (OR-PAM) will be discussed independently. Finally, the aforementioned resolution enhancement methods for photoacoustic microscopy will be critically evaluated, and the current challenges and future prospects of this technology will be summarized.

## 1. Introduction

**Figure 1:**
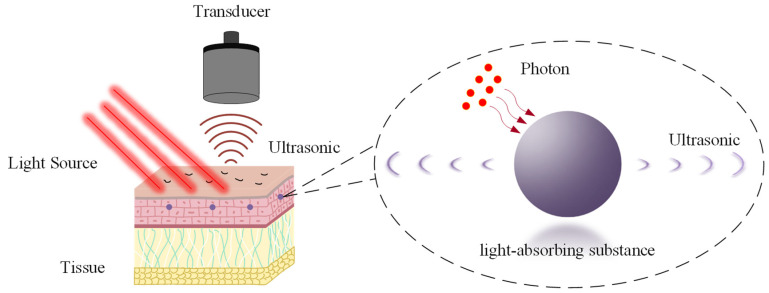
Schematic diagram illustrating the generation of the photoacoustic effect.

The photoacoustic effect was first described by Alexander Graham Bell in 1880 during his investigations into optical communication [1]. Subsequent research has explored this phenomenon extensively, leading to its application in diverse fields, including optical communications [2], photoacoustic spectroscopy [3], and photoacoustic imaging [4]. In particular, photoacoustic imaging has emerged as a powerful imaging modality with applications in both biomedical research and industrial inspection. A key advantage of photoacoustic imaging in the biomedical field stems from its unique imaging mechanism. Unlike modalities such as X-ray and ultrasound imaging [5,6], which rely on the scattering properties of substances within the imaged object, photoacoustic imaging exploits the absorption characteristics of chromophores in biological tissues or organic materials as its primary source of contrast. The absorption of light energy leads to localized increases in temperature, inducing microscopic thermoelastic expansion and contraction. These microscopic oscillations, in turn, generate macroscopic acoustic waves. Furthermore, photoacoustic imaging offers the advantages of being non-destructive, non-invasive, and biocompatible. In modern clinical medicine, photoacoustic imaging has demonstrated distinct advantages in resolution and imaging depth in applications such as blood oxygen saturation monitoring, vulnerable atherosclerotic plaque diagnosis, and multimodal ophthalmic imaging. Furthermore, regarding safety, photoacoustic imaging fully meets the requirements for human medical applications. Research by Michelle Heijblom et al. indicates that patients prefer photoacoustic imaging examinations due to their comfort, safety, and lack of side effects [7,8,9]. Therefore, due to its unique imaging mechanism and inherent advantages, photoacoustic imaging has the potential to become a valuable tool for biomedical imaging and detection. Figure 1 illustrates the principle of the photoacoustic effect.

**Figure 2:**
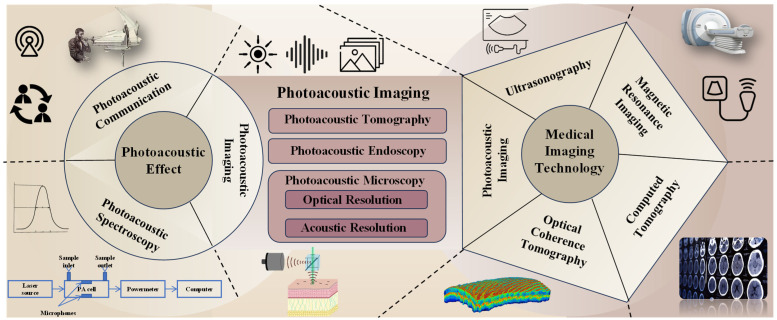
Photoacoustic imaging and its connections within the field of medical imaging technology.

With the development of related technologies, such as high-performance acoustic transducers and lasers, photoacoustic imaging gradually evolved into three research branches in the late 1990s: photoacoustic tomography, photoacoustic endoscopy, and photoacoustic microscopy [10]. Compared to the other two research branches, photoacoustic microscopy prioritizes resolution, especially lateral resolution, to achieve microscopic imaging capabilities, often at the expense of imaging depth and speed. Figure 2 depicts the classification of photoacoustic imaging within the broader field of medical imaging technologies.

During its initial development, photoacoustic microscopy, employing various high-performance acoustic transducers, demonstrated remarkable detection capabilities. With its micrometer-scale resolution, non-invasive nature, and sensitivity to optical properties, photoacoustic microscopy was recognized as a groundbreaking biomedical imaging modality that combined the advantages of both optical and acoustic imaging approaches. During this early period, the lateral resolution of photoacoustic microscopy was primarily determined by the bandwidth, central frequency, and focal spot size of the acoustic transducer. Consequently, these early photoacoustic imaging systems were designated as “acoustic-resolution photoacoustic microscopes (AR-PAMs)”, a term that distinguishes them from “optical-resolution photoacoustic microscopy (OR-PAM)”, a concept introduced by Konstantin Maslov et al. in 2008 [11]. Maslov’s research group utilized silicone oil to achieve a coaxial alignment of the optical and acoustic beams, thereby developing the first optical-resolution photoacoustic confocal microscopy system. Using this system, they successfully imaged the ear vasculature of nude mice with a lateral resolution of approximately 5 μm, significantly enhancing the imaging capabilities of photoacoustic microscopy. Thereafter, photoacoustic microscopy was broadly categorized into two main types: optical-resolution photoacoustic microscopy (OR-PAM) and acoustic-resolution photoacoustic microscopy (AR-PAM) [11].

The performance of photoacoustic imaging systems is assessed using several key metrics, including imaging depth, imaging speed, and resolution. Resolution is arguably the most critical parameter in the field of photoacoustic microscopy. Numerous methods and approaches have been developed to improve the resolution of photoacoustic microscopy, consequently expanding its range of applications. Beyond bolstering the research capabilities of photoacoustic imaging, this also allows for clinical applications in a range of finer-scale imaging tasks, including capillary blood oxygen saturation imaging and imaging of nuclear genetic material. This expands the clinical utility of photoacoustic microscopy, taking full advantage of the strengths of photoacoustic imaging. This paper presents a comprehensive discussion of the primary methods and recent advancements utilized to enhance the resolution of photoacoustic microscopy.

## 2. Principles of the Photoacoustic Effect

As discussed earlier, the photoacoustic effect utilizes the light absorption properties of materials to generate contrast and employs acoustic waves for signal detection and image formation. Therefore, the fundamental principle of photoacoustic imaging involves illuminating the target with pulsed laser light, which can be either unmodulated or modulated. The absorbed light energy then leads to a localized increase in temperature within the target. If the pulse duration of the laser is shorter than the thermal relaxation time of the target material [12], the following condition is met:

(1) $$t_{L}<\frac{d_{c}}{v_{s}}<\frac{d_{c}^{2}}{4\alpha_{\mathrm{th}}}$$

Here, $t_{L}$ represents the laser pulse width, $d_{c}$ denotes the characteristic length of thermal heterogeneity (attenuation constant of optical energy deposition), $v_{s}$ stands for the speed of sound, and $\alpha_{\mathrm{th}}$ signifies thermal diffusivity. When the laser pulse width is shorter than the thermal relaxation time, the absorbed energy primarily contributes to thermoelastic expansion of the medium. Therefore, periodic irradiation with short laser pulses induces periodic thermoelastic expansion, which generates acoustic pressure waves. These pressure waves then propagate outwards as acoustic waves. Conversely, if the laser pulse duration exceeds the thermal relaxation time, thermal diffusion dominates, leading to the establishment of a new thermal equilibrium within the medium. In this case, the efficiency of acoustic wave generation is significantly reduced, becoming negligible. When the conditions for photoacoustic signal generation are met, an ultrasonic transducer placed near the medium detects the generated acoustic waves. Subsequently, image reconstruction algorithms such as time-of-flight analysis are applied to the detected acoustic signals to create a photoacoustic image of the target medium.

### 2.1. Photoacoustic Equation

To further elucidate the underlying physical principles of photoacoustic imaging, a brief overview of the relevant theoretical framework is presented. Under thermal confinement conditions, the initial acoustic pressure $p_{0}$ is expressed as [12]:

(2) $$p_{0}=\frac{\beta T}{\kappa }$$

Here, β represents the coefficient of volumetric thermal expansion, T denotes the change in temperature, and κ signifies the isentropic compressibility.

(3) $$T=\frac{A_{e}}{\rho C_{V}}$$

$A_{e}$ denotes the specific or volumetric absorption coefficient, which also represents the optical energy deposition density. ρ represents the density. $C_{V}$ represents the isochoric specific heat capacity. The Grüneisen parameter Γ is defined as follows:

(4) $$\Gamma =\frac{\beta }{\kappa \rho C_{V}}$$

After introducing the Grüneisen parameter, the initial acoustic pressure can be rewritten as:

(5) $$p_{0}=\Gamma A_{e}$$

For short pulses, the thermodynamic equation is given by:

(6) $$\rho C_{V}\frac{\partial T(r,t)}{\partial t}=H(r,t)$$

H represents the thermal energy converted per unit volume and unit time, also known as the heating function. In this case, the photoacoustic equation can be written as:

(7) $$\left({𝛻}^{2}-\frac{1}{v_{s}^{2}}\frac{\partial^{2}}{\partial t^{2}}\right)p\left(r,t\right)=-\frac{\beta }{C_{P}}\frac{\partial H\left(r,t\right)}{\partial t}$$

The left-hand side of the equation describes the propagation of the generated acoustic waves, while the right-hand side represents the source term responsible for generating the photoacoustic signal, which is related to the absorbed optical energy and the thermoelastic properties of the medium.

### 2.2. Approaches for Resolution Characterization in Photoacoustic Microscopy

When designing and developing photoacoustic microscopy systems or conducting related research, a primary consideration is the method employed for characterizing the system’s resolution. Imaging resolution can be further categorized into two distinct components: lateral resolution and axial resolution. Subsequently, a concise overview of the measurement methods for both lateral and axial resolution will be presented.

#### 2.2.1. Lateral Resolution

Prior to the development of optical-resolution photoacoustic microscopy (OR-PAM), the lateral resolution of photoacoustic microscopy systems was primarily governed by the characteristics of the ultrasonic transducer, particularly its central frequency and numerical aperture. With the emergence of OR-PAM, the importance of achieving high lateral resolution has been increasingly recognized. Researchers have leveraged the principles of optical imaging to develop a variety of novel methods for characterizing lateral resolution. These experimental methods can be broadly categorized into three main approaches:

1. Knife-edge method

**Figure 3:**
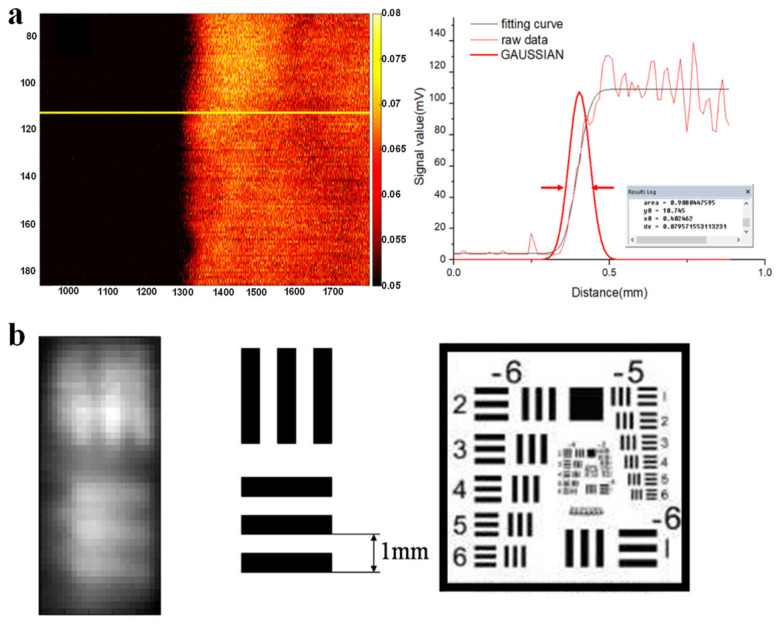
Experimental techniques for evaluating transverse resolution in photoacoustic imaging. (**a**) Knife-edge method [20]. (**b**) Resolution test target [21].

This method, referred to as the knife-edge method, involves imaging the sharp edge of a calibration target, such as the edge of a piece of black tape or a custom-fabricated photoacoustically sensitive plate. Once the edge has been imaged, a line profile perpendicular to the edge boundary is extracted. The edge spread function (ESF) is then calculated from the photoacoustic intensity profile along the extracted line. The line spread function (LSF) is subsequently obtained by calculating the derivative of the ESF. Finally, the full width at half maximum (FWHM) of the LSF is used as a measure of the system’s lateral resolution [13]. Figure 3a provides a schematic illustration of the knife-edge method.

2. Resolution test chart

The use of a resolution test chart is a standard approach for evaluating the resolution of imaging systems. For instance, the USAF 1951 resolution test chart is a widely employed standard. The test chart comprises multiple groups of bar patterns with varying spatial frequencies, arranged in decreasing order of size. Each group of patterns corresponds to a specific spatial resolution. If the bar patterns in a particular group are clearly resolved in the acquired image, the imaging system is deemed to have achieved the corresponding resolution [14,15]. Figure 3b presents a schematic diagram of the USAF 1951 resolution test chart.

3. Test landmarks

Directly imaging objects with well-defined dimensions provides a straightforward method for characterizing resolution. In photoacoustic imaging, calibration targets such as carbon fiber rods or gold nanoparticles are frequently utilized. This approach involves preparing a set of landmarks or fiducials with precisely controlled dimensions and then imaging them using the photoacoustic microscopy system. In some cases, LSF analysis may be employed to further refine the calibration. However, this approach can be challenging and potentially costly, as it requires the fabrication of fiducials with micro- or nanoscale features [16,17,18,19].

In addition to the three experimental methods described above, the approximate resolution of a photoacoustic microscopy system can be estimated using readily available specimens or objects with well-known dimensions. For example, a human hair has a diameter of approximately 50 μm, while a red blood cell typically has a diameter ranging from 6 to 8 μm. If these objects can be clearly imaged, it indicates that the system’s resolution is at least comparable to their respective dimensions [22]. Moreover, theoretical calculations can be employed to estimate the resolution, particularly when experimental measurements are impractical or unavailable. The following formula [23] can be used to calculate the transverse resolution of an OR-PAM system ($R_{O}$):

(8) $$R_{O}=n\frac{\lambda }{NA}$$

n represents the refractive index, λ represents the wavelength, and NA stands for the numerical aperture.

#### 2.2.2. Axial Resolution

Axial resolution ($R_{A}$) can be accurately measured experimentally by employing the fiducial marker method and analyzing the resulting line spread function (LSF) [24]. Theoretical calculations are also frequently employed to determine axial resolution. In theoretical analyses, the axial resolution can be directly calculated based on the bandwidth Δf and central frequency $f_{0}$ of the ultrasonic transducer [25], as expressed by the following formula:

(9) $$R_{A}\approx 0.88\frac{v_{s}}{\Delta f}=0.88\frac{v_{s}}{\eta f_{0}}\propto \frac{1}{f_{0}}$$

## 3. Methods for Improving Resolution in Photoacoustic Microscopy

PAM techniques are broadly categorized into two main types: AR-PAM and OR-PAM. This classification is based on whether the lateral resolution is primarily determined by acoustic or optical factors, respectively. In AR-PAM, the lateral resolution is primarily governed by the acoustic focal spot size of the ultrasonic transducer. In contrast, the lateral resolution of OR-PAM is primarily determined by the focal spot size of the excitation laser beam. Consequently, OR-PAM typically achieves a higher lateral resolution compared to AR-PAM. The axial resolution of both AR-PAM and OR-PAM is primarily determined by the bandwidth of the ultrasonic transducer; therefore, the two techniques generally exhibit similar axial resolution performance.

**Figure 4:**
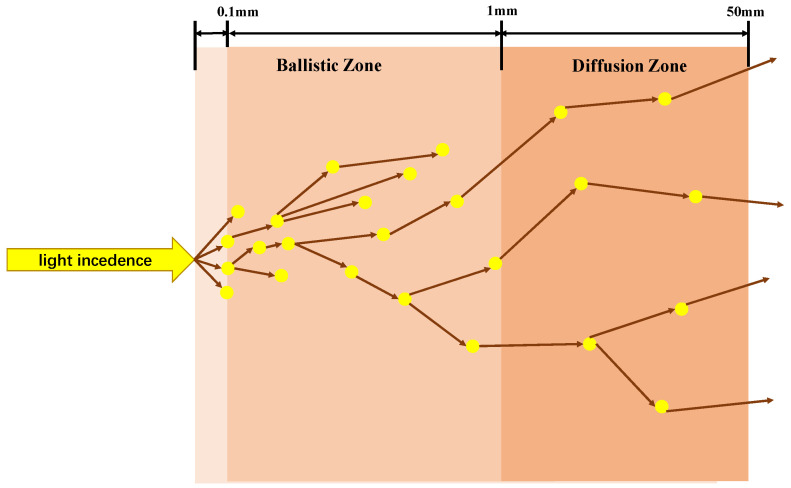
Schematic representation of photon propagation in biological tissue.

While optical-resolution photoacoustic microscopy (OR-PAM) provides enhanced lateral resolution, it typically exhibits a more limited imaging depth compared to acoustic-resolution photoacoustic microscopy (AR-PAM). Consider, for example, the imaging of human skin. The behavior of photons within the tissue varies significantly depending on the penetration depth following laser irradiation. Within one optical mean free path, photons can be assumed to propagate along their initial trajectories without scattering events. In biological tissues, the optical mean free path is typically on the order of 0.1 mm. Photons that travel within one optical mean free path are referred to as ballistic photons, while those that travel within approximately ten optical mean free paths are termed snake photons. Within this range, the trajectories of snake photons deviate only slightly from their initial paths. Consequently, the region within approximately ten optical mean free paths (approximately 1 mm in biological tissues) is often referred to as the ballistic regime. Beyond the ballistic regime, as photons penetrate deeper into the tissue, the scattering probability increases substantially. In this highly scattering regime, both the spatial resolution and the signal-to-noise ratio of photoacoustic imaging degrade significantly. The maximum penetration depth of light in biological tissues is typically limited to approximately 5 cm. Beyond this depth, the photoacoustic signal becomes negligible [26]. Figure 4 illustrates the different regimes of photon propagation in biological tissue. This analysis suggests that, in principle, the maximum imaging depth achievable with OR-PAM is typically less than that of AR-PAM.

**Figure 5:**
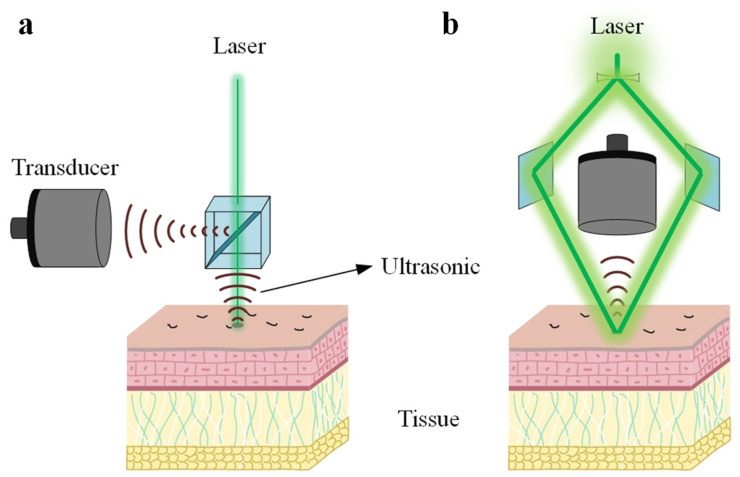
Schematic diagram of two types of photoacoustic imaging. (**a**) Optical-resolution photoacoustic microscopy (OR-PAM). (**b**) Acoustic-resolution photoacoustic microscopy (AR-PAM).

**Figure 6:**
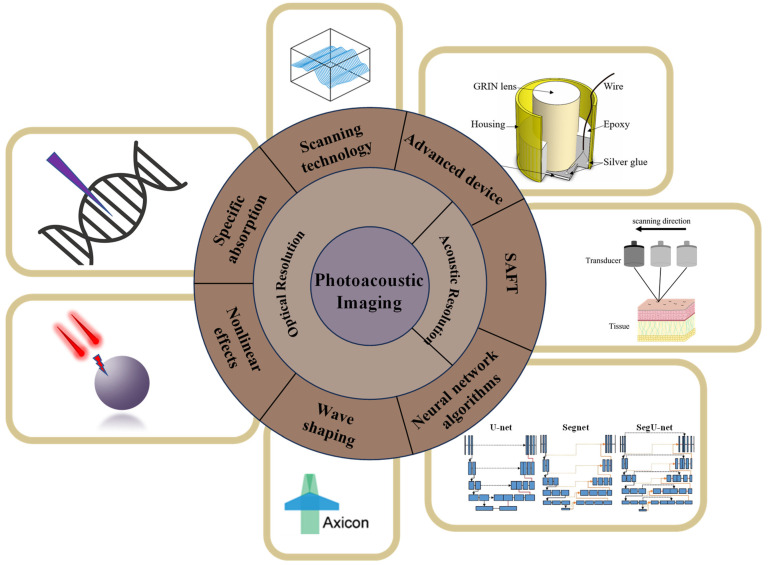
Overview of resolution enhancement methods in photoacoustic microscopy [20,27,28,29].

Both optical-resolution and acoustic-resolution photoacoustic microscopy techniques are well suited for specific applications. Recent years have seen substantial progress in both OR-PAM and AR-PAM, driven by the collaborative efforts of researchers worldwide. Figure 5a,b depict the schematic diagrams of the operational principles of OR-PAM and AR-PAM, respectively. The primary difference between OR-PAM and AR-PAM lies in whether the lateral resolution is determined by optical or acoustic factors, respectively. Subsequent sections will provide a detailed discussion of the methods and advancements aimed at enhancing the resolution of both AR-PAM and OR-PAM. Figure 6 presents a comprehensive overview of the various techniques employed to improve the imaging resolution of photoacoustic microscopy, as discussed throughout this review.

### 3.1. Methods and Advances in Improving Resolution of AR-PAM

Photoacoustic microscopy began attracting increasing interest from researchers with the emergence and development of photoacoustic imaging technology. As early as the 1990s, the research group led by C.G.A. Hoelen developed a photoacoustic microscopy system capable of acquiring three-dimensional (3D) images of tissues and blood vessels. Their system utilized a polyvinylidene fluoride (PVDF)-based planar transducer and incorporated several image processing algorithms, including the synthetic aperture focusing technique (SAFT), to enhance image quality within a specific depth range. Using chicken breast tissue as a phantom to mimic the acoustic properties of biological tissue, the researchers achieved an imaging depth of 5 mm. Theoretical calculations based on the transducer’s size and central frequency indicated a lateral resolution of 200 μm and an axial resolution of 10 μm [30]. This review represents one of the earliest demonstrations of AR-PAM, and the developed imaging system provides valuable insights for the design of future photoacoustic microscopy systems. In the subsequent sections, we will present a detailed overview of various methods employed to enhance the resolution of AR-PAM systems (summarized in Table 1).

#### 3.1.1. Transducer Optimization

This section focuses on the most fundamental approaches for enhancing resolution in AR-PAM, including the utilization of ultrasonic transducers with high numerical apertures (NAs), high central frequencies, and innovative designs. These methods represent the most straightforward strategies for improving resolution and serve as a benchmark for evaluating the performance of more advanced techniques. A comparison of the achievable lateral and axial resolutions using these fundamental approaches provides insights into the performance limits of conventional AR-PAM systems.

During the initial development of PAM, drawing upon established principles from ultrasound imaging, researchers recognized that the utilization of high-frequency, wide-bandwidth, and high-numerical-aperture (NA) ultrasonic transducers could enhance imaging resolution. Continuous advancements in ultrasonic transducer technology have led to significant improvements in imaging performance, thereby expanding the capabilities of PAM. In 2019, Moothanchery et al. developed a high-speed photoacoustic microscopy system based on galvo-mirror scanning. The researchers evaluated the performance of two different transducers: one with a central frequency of 50 MHz and another with a central frequency of 75 MHz. A 6 μm carbon fiber rod was used as a target for imaging, and the acquired photoacoustic data were reconstructed using the maximum intensity projection (MIP) method. The lateral resolution was quantified as the full width at half maximum (FWHM) of the photoacoustic signal generated by the carbon fiber rod. The experimental results demonstrated that the 50 MHz transducer achieved a lateral resolution of 84 μm and an axial resolution of 27 μm, while the 75 MHz transducer achieved a lateral resolution of 53 μm and an axial resolution of 18 μm [31]. To assess the imaging depth, the researchers imaged chicken breast tissue embedded with black needles. The measured imaging depth was 2.7 mm for the 50 MHz transducer and 1.8 mm for the 75 MHz transducer. Using their custom-built AR-PAM system, the researchers demonstrated that AR-PAM offers a significant advantage over OR-PAM in terms of imaging depth. They also emphasized the fundamental trade-off between spatial resolution and imaging depth that is inherent to AR-PAM systems. Finally, they successfully demonstrated the high-speed scanning capabilities and practical utility of their system by performing in vivo imaging experiments on mice.

**Figure 7:**
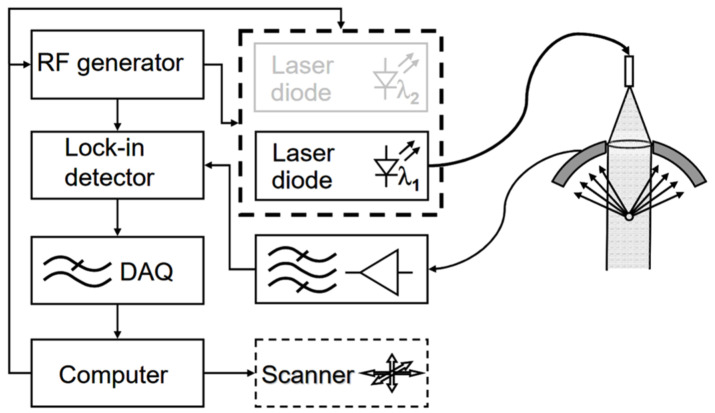
Schematic diagram of the continuous-wave photoacoustic microscope developed by Konstantin Maslov and Lihong V. Wang [18].

In addition to utilizing commercially available transducers, numerous research groups have actively pursued the development of novel transducer designs to optimize their structure and improve their performance for photoacoustic imaging. These research efforts have provided valuable insights and knowledge that have advanced the design and fabrication of specialized transducers optimized for photoacoustic imaging applications. In 2007, Maslov and Wang developed a continuous-wave photoacoustic imaging system that did not rely on optical focusing and instead utilized a low-intensity, amplitude-modulated laser diode as the excitation source. They incorporated a novel bowl-shaped ultrasonic transducer (Figure 7) to achieve acoustic focusing and facilitate image formation [18]. Based on the definition of numerical aperture (NA): NA = n∙sin(α), the bowl-shaped transducer exhibits a larger NA than conventional planar transducers. Moreover, this design enables a more compact and coaxially aligned configuration of the photoacoustic imaging system. Experimental evaluation of the system’s resolution, using a USAF 1951 resolution test chart, demonstrated a lateral resolution of 600 μm and an axial resolution of 700 μm. Imaging experiments performed on rabbit leg vasculature showed that the system could achieve an imaging depth of 3 mm. Although the resolution of this system may not have been comparable to that of the most advanced AR-PAM systems available at the time, the innovative system configuration and transducer design provided a new perspective and approach for photoacoustic imaging.

**Figure 8:**
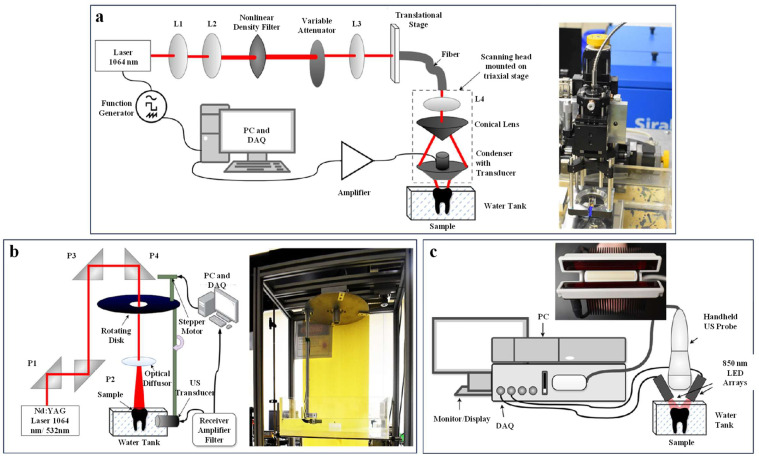
Photoacoustic imaging system for dental imaging [34]. (**a**) Acoustic-resolution photoacoustic microscope using a 1064 nm laser. (**b**) Circular photoacoustic tomography system operating at 532 nm and 1064 nm wavelengths. (**c**) Linear-array-based photoacoustic tomography (PAT) system operating at a 850 nm wavelength.

In addition to optimizing conventional transducer parameters such as central frequency and numerical aperture, employing specialized designs to improve transducer directivity can further enhance the resolution of photoacoustic microscopy systems. In 2003, Kolkman et al. developed a piezoelectric dual-ring transducer specifically designed for photoacoustic imaging. The transducer comprises two concentric ring electrodes with a piezoelectric material layer sandwiched between them. An optical fiber, which delivers the excitation light pulses, is positioned at the center of the transducer [32]. The dual-ring transducer, characterized by a small opening angle, exhibits a relatively low numerical aperture. Despite its low NA, the transducer demonstrates strong acoustic focusing capability and high directivity. These characteristics enable the transducer to achieve high spatial resolution while simultaneously minimizing side lobes and artifacts that are often associated with high-NA transducers. The researchers demonstrated the imaging system’s ability to visualize wrist blood vessels with diameters ranging from 0.03 mm to 1 mm. In 2024, Kim et al. introduced a novel UV-transparent ultrasonic transducer (UV-TUT) for photoacoustic imaging applications. With a system numerical aperture of 0.38 NA and a UV excitation wavelength of 266 nm, they achieved a spatial resolution of 0.47 ± 0.03 μm [33]. This UV-TUT-based PAM system is particularly well-suited for label-free imaging of DNA and RNA, owing to its high spatial resolution and the strong UV absorption of these biomolecules. Recent research in acoustic-resolution photoacoustic microscopy has increasingly focused on practical applications and clinical translation. In a recent study, Periyasamy et al. demonstrated the potential of photoacoustic microscopy for dental diagnostics (Figure 8) [34]. Their system, which incorporated a focused acoustic lens, achieved a lateral resolution of 130 μm and an axial resolution of 57 μm for dental imaging. With a scanning speed of 10 Hz, the system demonstrates sufficient speed for many practical applications in dentistry.

Furthermore, the optical path design and the relatively large size of acoustic components in conventional acoustic-resolution photoacoustic microscopy have hindered its clinical translation.

#### 3.1.2. Synthetic Aperture Focusing Technique (SAFT)

**Figure 9:**
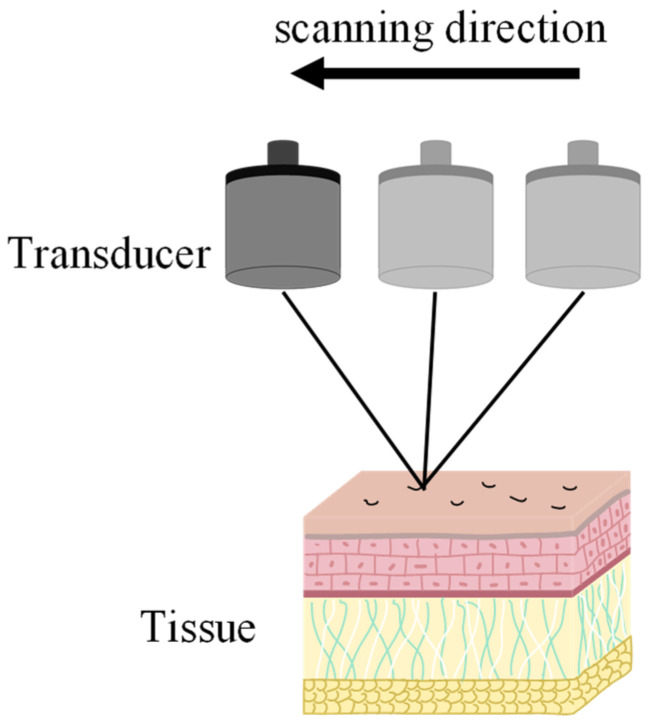
Schematic diagram of the synthetic aperture focusing technique.

The synthetic aperture focusing technique (SAFT) is a signal processing method used in ultrasound imaging to effectively increase the numerical aperture (NA) of the imaging system [40,41]. SAFT, which has its roots in synthetic aperture radar (SAR) [42,43], operates on the principle of synthesizing a larger aperture transducer by either scanning a single-element transducer or employing a transducer array. Subsequent signal processing techniques are then applied to reconstruct an image with enhanced lateral resolution (Figure 9).

**Figure 10:**
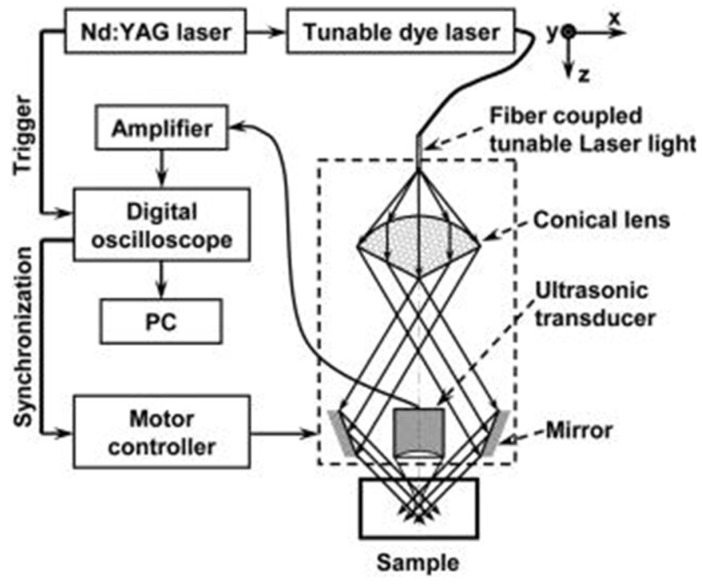
Schematic diagram of a dark-field illumination photoacoustic imaging system [49].

PAM, with its inherent point-by-point scanning acquisition mode, is particularly amenable to the implementation of the synthetic aperture focusing technique (SAFT). Consequently, the main drawback of applying SAFT in PAM is the increased computational burden associated with the signal processing. In 2006, Meng-Lin Li et al. conducted photoacoustic imaging experiments using SAFT and successfully demonstrated its significant contribution to resolution enhancement using a carbon fiber phantom. Their results showed a lateral resolution improvement, achieving a resolution of 46 μm to 53 μm. They also performed in vivo imaging of a rat scalp, successfully visualizing the microvasculature [36]. Later that year, the same group further refined the SAFT implementation by incorporating a correlation-weighted approach. This modification enhanced the resolution outside the focal zone while simultaneously suppressing side lobes and improving the signal-to-noise ratio (SNR) [37]. In another study from the same year, the research group employed a high-bandwidth, high-numerical-aperture (NA) transducer, achieving a lateral resolution of 45 μm and an axial resolution of 15 μm. They demonstrated the capabilities of this system by performing imaging experiments on rat dorsal blood vessels and human palms [35]. It is worth noting that the research group frequently employed dark-field illumination (Figure 10), a technique commonly used in AR-PAM systems. Although dark-field illumination does not directly improve the spatial resolution, it can enhance imaging depth by reducing the influence of strong superficial reflections. This contributes to highlighting the inherent advantage of AR-PAM in achieving greater imaging depths [44,45,46,47,48,49].

In 2023, Thomas et al. proposed an energy-compensated SAFT-based photoacoustic imaging method that accounts for acoustic attenuation by incorporating a compensation algorithm. This approach demonstrated a 5% improvement in both axial and lateral resolution [38]. SAFT can also be implemented with array-based transducers. However, current limitations in the fabrication of high-frequency ultrasonic transducer arrays restrict their widespread use in PAM, where high central frequencies are essential for achieving high resolution. Consequently, array transducers are more commonly employed in applications such as photoacoustic computed tomography (PACT), where the requirements for transducer frequency are less demanding [50,51,52].

SAFT can be readily integrated with high-performance transducers, requiring only additional computational time for post-processing to generate images with enhanced resolution. Although this additional processing time may impact real-time imaging capabilities, the effect is generally negligible compared to the time required for mechanical scanning in conventional PAM systems. While SAFT technology can be valuable in some clinical applications based on scanning imaging, its relatively slow scanning speed may present a limitation for real-time imaging capabilities.

#### 3.1.3. Neural Network Algorithms

**Figure 11:**
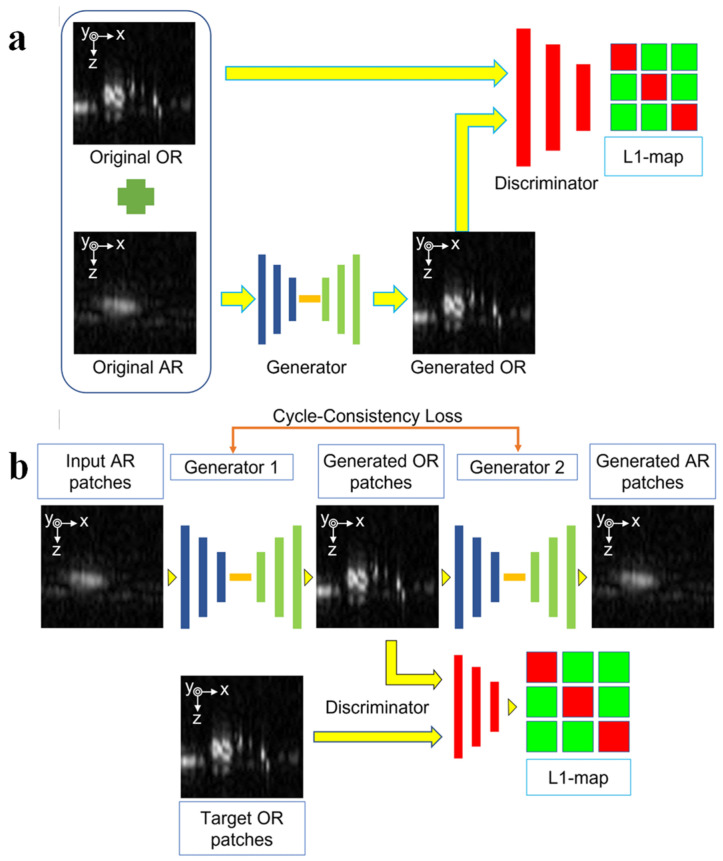
Sample extraction during 3D training using (**a**) cGAN and (**b**) CycleGAN models, respectively [36].

The concept of neural network algorithms was first introduced in the mid-20th century. Driven by the rapid advancements in computer hardware in the early 21st century, neural network algorithms have experienced a resurgence, leading to the development of various architectures, including deep neural networks, convolutional neural networks, and generative adversarial networks, among others [53,54,55,56,57]. In 2023, Le et al. [39] investigated the use of two types of generative adversarial networks (GANs) for photoacoustic image reconstruction: a semi-supervised conditional GAN (cGAN) and an unsupervised cycle-consistent GAN (CycleGAN). Their study aimed to compare the performance of these two GAN architectures in enhancing photoacoustic image quality (Figure 11) [39]. They developed a photoacoustic imaging system based on a microelectromechanical systems (MEMS) scanning mirror. This system could operate in two modes: OR-PAM, which utilized a single-mode fiber for optical excitation, and AR-PAM, which employed a multimode fiber. The cGAN training process requires paired data, where each AR-PAM image is paired with a corresponding OR-PAM image that serves as the ground truth label. In contrast, CycleGAN does not require paired data for training. Instead, it leverages cycle consistency to enforce the reversibility of the image transformation process. This means that an image transformed from AR-PAM to OR-PAM and then back to AR-PAM should ideally be identical to the original AR-PAM image. cGAN requires less training time and offers faster image generation, while CycleGAN, although requiring more extensive training, can generate images of higher quality. The researchers demonstrated that, by utilizing the trained GANs, it is possible to enhance the image quality of AR-PAM to a level comparable to that of OR-PAM. The structural similarity index (SSIM) between the GAN-generated images and the corresponding OR-PAM images reached 0.97 for cGAN and 0.94 for CycleGAN, indicating a high degree of fidelity. Aside from the data requirements and computational resources needed for training the GANs, the time required for image processing and generation using a trained network is negligible and does not significantly affect real-time imaging performance. Therefore, with the rapid development of computer technology and GPU computing power, a growing number of research achievements utilizing neural networks to enhance photoacoustic imaging are emerging. This method can be applied to any scenario, including clinical use, and has become a hotspot in modern photoacoustic imaging development.

The primary methods for enhancing the spatial resolution of AR-PAM can be categorized into the three approaches discussed above. A major challenge in advancing AR-PAM technology lies in the development of high-performance acoustic transducers. In addition to the direct improvements in imaging performance resulting from advances in transducer technology, various signal processing techniques from the field of ultrasound imaging can be readily adapted and applied to AR-PAM. Furthermore, the integration of deep learning and neural network techniques holds great promise for further enhancing the capabilities of AR-PAM and expanding its applications in various imaging fields. Table 2 presents the clinical usability and the associated advantages and disadvantages of acoustic-resolution photoacoustic microscopy.

### 3.2. Methods and Advancements in Resolution Enhancement for Optical-Resolution Photoacoustic Microscopy

The development of OR-PAM marked a significant turning point in the advancement of PAM, ushering in a period of rapid progress. In 2008, Maslov’s group developed a confocal PAM system that employed tightly focused optical bright-field illumination and a 75 MHz ultrasonic transducer with acoustic focusing, achieving a lateral resolution of 5 μm and an axial resolution of 15 μm [11]. This system achieved a maximum imaging depth of over 0.7 mm and demonstrated a lateral resolution of 5 μm for in vivo imaging of blood vessels. This pioneering work established the foundation and fundamental principles of OR-PAM, demonstrating the potential of leveraging the strong focusing capability of optics to significantly improve the lateral resolution of PAM. This breakthrough was particularly important for microscopic imaging applications, emphasizing the key advantages of PAM: high spatial resolution enabled by optical focusing and relatively deep penetration depth provided by acoustic detection. As a result, OR-PAM has emerged as a major research focus within the field of PAM, with ongoing efforts to develop innovative methods for further enhancing its spatial resolution (Table 3).

#### 3.2.1. Employing High-Performance Optical Objectives and Transducers

**Figure 12:**
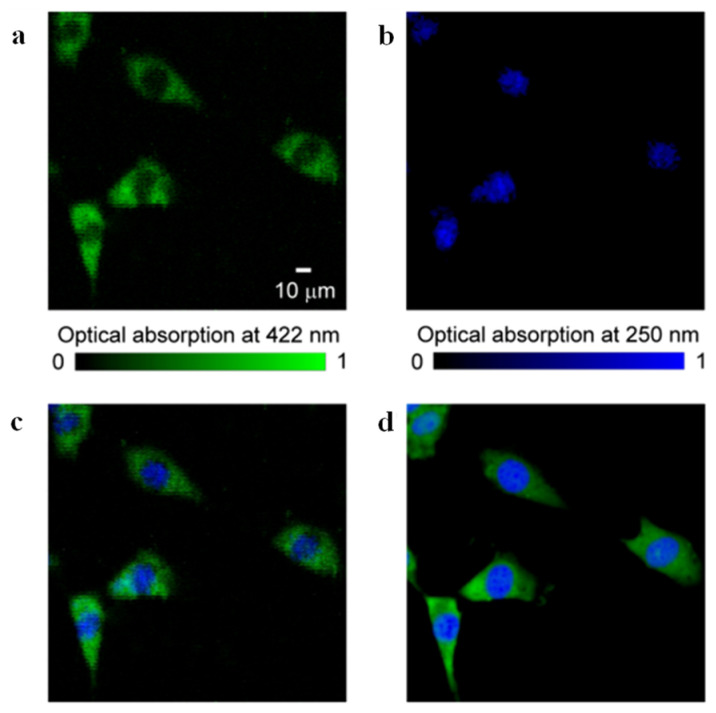
Photoacoustic and fluorescence microscopy images of fibroblasts [60]. (**a**) Label-free photoacoustic microscopy image of a fixed but unstained fibroblast acquired at a wavelength of 422 nm. (**b**) Label-free photoacoustic microscopy image acquired at a wavelength of 250 nm. (**c**) Overlay of images (**a**,**b**). (**d**) Fluorescence microscopy image of the cell, with mitochondria shown in green and the nucleus in blue.

In optical microscopy, the use of high-NA objective lenses is a standard method for achieving tighter focusing of the illumination beam. Advances in lens fabrication technology have led to the widespread availability of high-NA objective lenses. In PAI, which leverages both optical excitation and acoustic detection, the use of high-NA objective lenses to achieve tighter focusing of the excitation light is also a fundamental approach for improving spatial resolution [85,86,87]. In 2009, Bost et al. developed a PAM platform that incorporated transducers fabricated from two different piezoelectric materials. Using a high-NA objective lens and a 400 MHz ultrasonic transducer, and employing carbon and iron oxide microparticles as phantoms, they demonstrated a lateral resolution of 3.6 μm with their system [58]. In 2010, Maslov et al. integrated a 0.85 NA, 125 MHz ultrasonic transducer with a 0.6 NA objective lens. Employing a precise photoacoustic confocal configuration, they achieved a lateral resolution of 0.55 μm, approaching the diffraction-limited focusing spot size of a 532 nm laser, and further refined the concept of OR-PAM [59]. Later that year, the same research group explored the use of even higher-NA objective lenses to further improve the spatial resolution. Employing a 1.23 NA objective lens and measuring the FWHM of the photoacoustic signal generated by gold nanospheres (which can be approximated by a Bessel function), they achieved a lateral resolution of 220 ± 20 nm, approaching the theoretical diffraction limit [23]. These two studies highlight the substantial impact of high-NA objective lenses on achieving high spatial resolution in OR-PAM. In 2012, Zhang et al. employed two custom-designed high-NA objective lenses to perform dual-wavelength PAI. A 0.6 NA objective lens with a 422 nm excitation wavelength was used to image the cytoplasm of fixed fibroblasts, while a 0.4 NA objective lens with a 250 nm excitation wavelength was used to specifically image the cell nucleus [60]. Figure 12 demonstrates that this dual-wavelength PAI approach can achieve a level of spatial resolution comparable to that of fluorescence microscopy.

**Figure 13:**
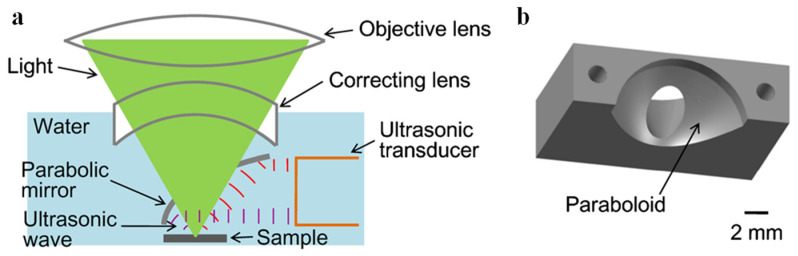
Photoacoustic signal reflection device used by Chi Zhang et al. in 2012 [61]. (**a**) Schematic of the core system. Acoustic focusing is achieved by the parabolic mirror, which has a central conical hole for light delivery. (**b**) 3-D model of the parabolic mirror.

In early photoacoustic imaging studies, high spatial resolution was primarily achieved using transmission-mode configurations. This is because reflection-mode configurations require both the optical illumination and acoustic detection paths to be located on the same side of the sample and to be coaxially aligned, which poses significant challenges for system design. It is challenging to simultaneously achieve high NAs for both the optical focusing and acoustic detection components in a reflection-mode configuration. Consequently, the lateral resolution of earlier reflection-mode PAM systems was typically limited to values greater than 2 μm. In 2012, Zhang et al. addressed this challenge by employing a custom-designed parabolic mirror to realize reflection-mode PAM. The parabolic mirror focused and reflected the generated photoacoustic waves, allowing a larger portion of the acoustic signal to be collected without interfering with the optical focusing path (Figure 13) [61]. Moreover, they used a 0.63 NA objective lens for optical focusing, with an excitation wavelength of 532 nm. Using a resolution test chart, they achieved a lateral resolution of 0.5 μm with this system. Comparison of imaging results obtained from mice demonstrated that this subwavelength-resolution PAM system enabled superior visualization and analysis of capillaries.

**Figure 14:**
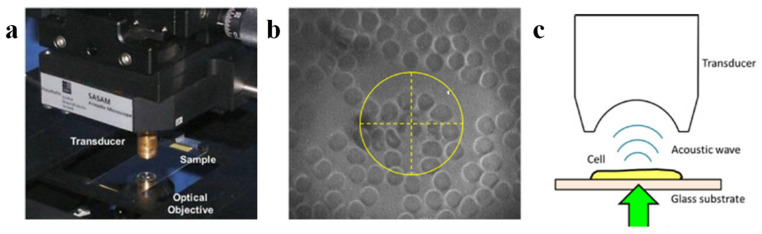
Photoacoustic imaging system developed by Eric M. Strohm et al. [62]. (**a**) Photograph of the system configuration. (**b**) Optical view of stained blood cells. (**c**) Schematic illustration of photoacoustic wave propagation.

In addition to employing high-NA objective lenses and ultrasonic transducers, utilizing high-frequency transducers is another common strategy for improving spatial resolution in PAM. In 2016, Strohm et al. demonstrated single-cell imaging capabilities using a 1 GHz ultrasonic transducer and a 0.45 NA objective lens to image stained neutrophils and lymphocytes, achieving a lateral resolution of 1 μm (Figure 14) [62].

**Figure 15:**
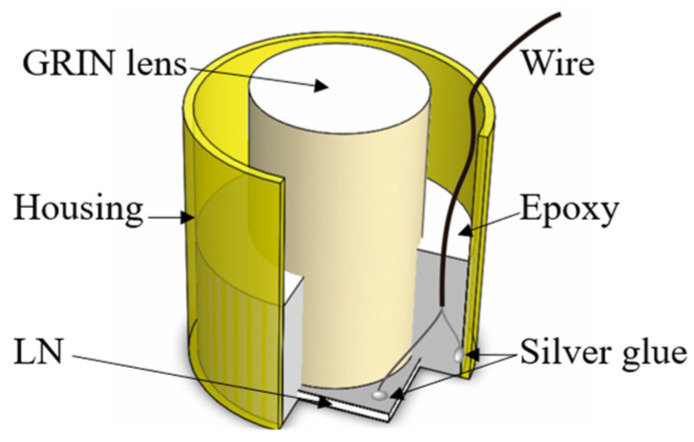
Photoacoustic probe incorporating a GRIN lens [20].

Miniaturization has become an increasingly important trend in the development of PAM systems for practical applications. In 2018, Chen et al. developed a handheld PAM probe with dimensions of 22 × 30 × 13 mm and a weight of 20 g. This probe integrated a 0.1 NA objective lens and a 10 MHz ultrasonic transducer. Using the knife-edge method, they achieved a lateral resolution of 3.8 μm and an axial resolution of 104 μm with this probe. They performed imaging experiments on various biological tissues, including mouse ears, to demonstrate the probe’s capabilities [63]. Compared to the PAM systems discussed earlier, the relatively low NA of the objective lens and the low center frequency of the ultrasonic transducer in this probe resulted in a lower spatial resolution. In 2023, Lin et al. developed a miniaturized multifunctional optoelectronic probe that achieved a lateral resolution of 80 μm. This probe integrated advanced acoustic and optical components, including a transparent ultrasonic transducer and a GRIN (gradient refractive index) lens. The transparent piezoelectric material, along with an outer diameter of only 4 mm and a transducer center frequency of 47 MHz, enabled a compact probe design. Significantly, this study represented the first reported use of a GRIN lens in a miniaturized PAM probe, contributing to a further reduction in the probe’s size (Figure 15) [20].

The studies discussed above demonstrate that the use of high-frequency ultrasonic transducers and high-NA objective lenses is crucial for achieving high spatial resolution in PAM. However, both high-frequency transducers and high-NA objective lenses have inherent limitations. High-frequency transducers generally exhibit reduced penetration depth and a smaller depth of focus, while high-NA objective lenses are associated with a shallow depth of field. Moreover, several engineering challenges related to the design, fabrication, and integration of these components into PAM systems still need to be addressed. Therefore, in addition to continued advancements in device technology, researchers in the field of PAM must actively investigate and develop novel components and imaging methodologies specifically tailored to the unique requirements of PAI. In clinical applications, it is also necessary to consider whether high-numerical-aperture, highly focused objective lenses might lead to excessive optical energy density.

#### 3.2.2. Advancements in Scanning Devices and Techniques

**Figure 16:**
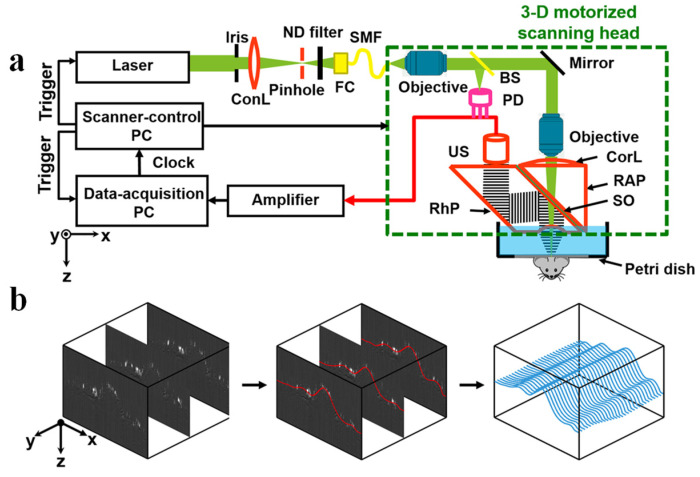
Profiling-scanning photoacoustic microscope [27]. (**a**) Schematic of a continuous three-dimensional motorized profiling-scanning optical-resolution photoacoustic microscope. (**b**) Flowchart of the scanning algorithm.

Scanning mechanisms play a critical role in PAM systems. Commonly employed scanning methods include motorized translation stages and MEMS-based scanning mirrors. Motorized stages typically provide higher precision and stability but are relatively slow, whereas MEMS-based mirrors offer faster scanning speeds but may have lower precision and higher costs. Advances in MEMS technology and the growing demand for high-speed PAM imaging in practical applications have led to the increased adoption of MEMS-based scanning mirrors, although both motorized stages and MEMS-based mirrors have their specific advantages and disadvantages, making them suitable for different applications [17,24,88]. In 2022, Cao et al. developed a novel PAM system that incorporated various advanced imaging techniques, including ultraviolet (UV) lasers and deep learning algorithms. They also implemented real-time 3D profiling scanning, an advanced scanning method particularly well suited for PAI of samples with non-planar surfaces [64]. This study focused on imaging the genetic material within bone tissue and achieved a lateral resolution of 820 nm, as measured using black tape as a phantom for resolution characterization. Real-time 3D profiling scanning utilizes time-of-flight measurements to acquire real-time information about the sample’s surface topography. A high-precision Z-axis stepper motor or other actuator is employed to dynamically adjust the position of the optical focus, maintaining the sample surface within the focal plane and thus optimizing the spatial resolution of the imaging system. Real-time 3D profiling scanning not only facilitates high-precision imaging of samples with complex surface geometries but also expands the application range of high-NA objective lenses with limited depths of field, thereby improving the achievable spatial resolution in PAM [27,65]. Figure 16 represents the real-time 3D contour scanning photoacoustic imaging system developed by Chenghung Yeh et al. [27]. The improvements to the scanning devices or methods fully consider the scenarios of irregular object scanning in clinical applications and have the potential to further reduce the size of photoacoustic scanning devices, providing significant reference and guidance for clinical applications.

#### 3.2.3. Exploiting the Specific Absorption Properties of Light Waves

In PAI, image contrast is generated by the differential absorption of light by various components within the sample. Similar to how the diverse colors of objects in the natural world arise from their varying light reflection properties, each material also possesses a unique optical absorption spectrum. Consequently, by selecting specific excitation wavelengths, it is possible to selectively enhance the PA signal from particular targets within the sample. Furthermore, integrating multiple excitation wavelengths into a single PAI system can significantly broaden its range of applications. Alternatively, exogenous contrast agents can be introduced to enhance the optical absorption of specific targets within the sample at a particular wavelength, thereby increasing the generated PA signal amplitude and improving both image contrast and spatial resolution. Numerous studies have investigated the use of various contrast agents to enhance the PA signal amplitude or improve the efficiency of photothermal conversion. Table 4 provides a list of commonly used PA contrast agents, along with their key properties and applications [66].

**Figure 17:**
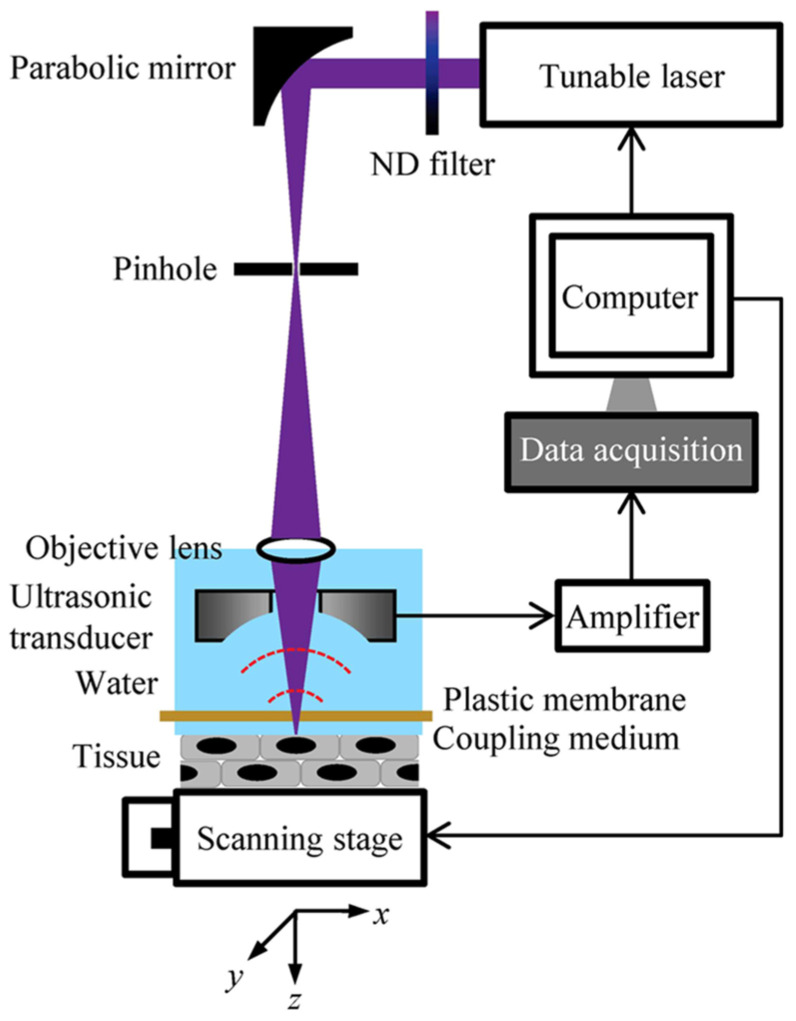
The ultraviolet photoacoustic imaging system built by the research team of Da-Kang Yao [68].

This review has focused on several commonly employed methods for enhancing spatial resolution in PAM. In 2010, Yao et al. developed a technique for imaging cells based on the strong UV absorption of RNA and DNA. This technique employed a low-NA (0.1) objective lens and a 50 MHz ultrasonic transducer, achieving a lateral resolution of 700 nm, as determined using black polystyrene microspheres as a resolution phantom [67]. In addition to the strong UV absorption exhibited by nucleic acids, UV light also offers superior focusing capability compared to longer wavelength lasers due to its shorter wavelength, which allows for a smaller diffraction-limited spot size. In 2012, the same group successfully imaged DNA and RNA within the nuclei of mouse ear cells using UV excitation, achieving a lateral resolution of 700 nm [68]. Figure 17 depicts the UV-PAM system developed by the research group. The use of UV light for PAI of nucleic acids continues to be an active area of research. In 2021, Baik et al. developed a high-speed reflection-mode UV-PAM system that employed a MEMS-based scanning mechanism. This system enabled high-speed imaging of unlabeled cell nuclei with a spatial resolution of 1.2 μm [69].

**Figure 18:**
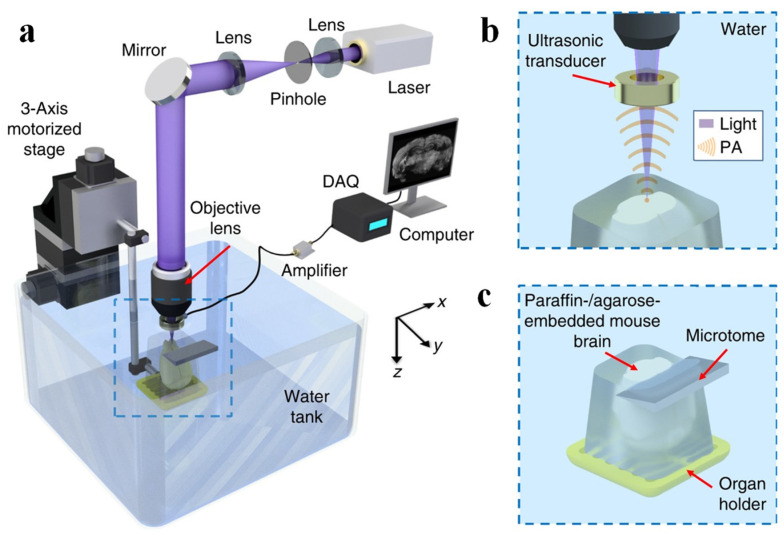
Schematic of the mPAM system for whole-organ imaging and sectioning. (**a**) Schematic diagram of the ultraviolet photoacoustic imaging system. (**b**) Close-up of the blue dashed region during imaging. (**c**) Close-up of the blue dashed region during sectioning. Imaging surface is being sectioned [71].

Gold nanoparticles (GNPs) are widely used as contrast agents in PAI to enhance the PA signal. In 2011, Rao et al. leveraged the nonlinear optical absorption properties of GNPs to improve photothermal conversion efficiency, thereby increasing the PA signal amplitude from red blood cells. This study employed a 0.6 NA objective lens and achieved a lateral resolution of 0.23 μm ± 0.03 μm, as determined by measuring the LSF of GNPs. The study also highlighted the system’s high-speed scanning capabilities [70]. In 2017, Wong et al., motivated by the potential for tissue damage and distortion caused by conventional microtomy techniques, developed a system that integrated automated tissue sectioning with PAM imaging. They employed excitation wavelengths of 266 nm and 420 nm, using the former for imaging DNA/RNA and the latter for imaging cytochromes. The system achieved a lateral resolution of 0.91 μm and an axial resolution of 20 μm. This approach enabled 3D PAI of organs such as the mouse brain and kidney (Figure 18) [71]. Later that year, the same research group leveraged the high spatial resolution afforded by UV excitation and the strong UV absorption of cell nuclei to image human breast cancer tissues. The lateral resolution of the imaging system was determined to be 330 nm, as calibrated using GNPs [72]. These studies highlight the potential of leveraging the specific absorption properties of biological molecules in the UV range, in conjunction with UV excitation, to achieve high spatial resolution in PAM.

**Figure 19:**
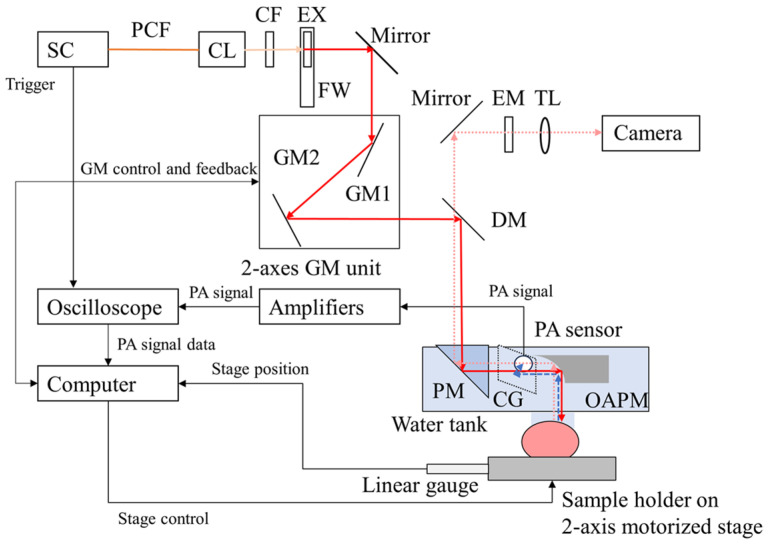
Wavelength-tunable photoacoustic imaging system using a supercontinuum laser source [73].

Indeed, different materials exhibit distinct optical absorption spectra, with peak absorption occurring at specific wavelengths. For example, in 2024, Hirasawa et al. developed a wavelength-tunable PAM system that utilized a supercontinuum laser source capable of generating light from 500 nm to 2200 nm. By incorporating tunable bandpass filters, they were able to perform PAI at different wavelengths. The authors demonstrated imaging of hemoglobin using 550–600 nm excitation light and near-infrared contrast agents using 748–789 nm excitation light, achieving a maximum lateral resolution of 3.8 μm. Figure 19 presents a schematic diagram of the wavelength-tunable PAM system developed by Hirasawa et al. [73]. This type of wavelength-tunable PAM system represents a significant advance in the field, as it enables imaging of a broader range of targets and contrast agents and has the potential to expand the applications of PAM in various fields. In clinical applications, it is compatible with other methods, and molecular probe technology based on this principle is currently one of the development trends in photoacoustic microscopy.

#### 3.2.4. Acoustic or Optical Wavefront Shaping

**Figure 20:**
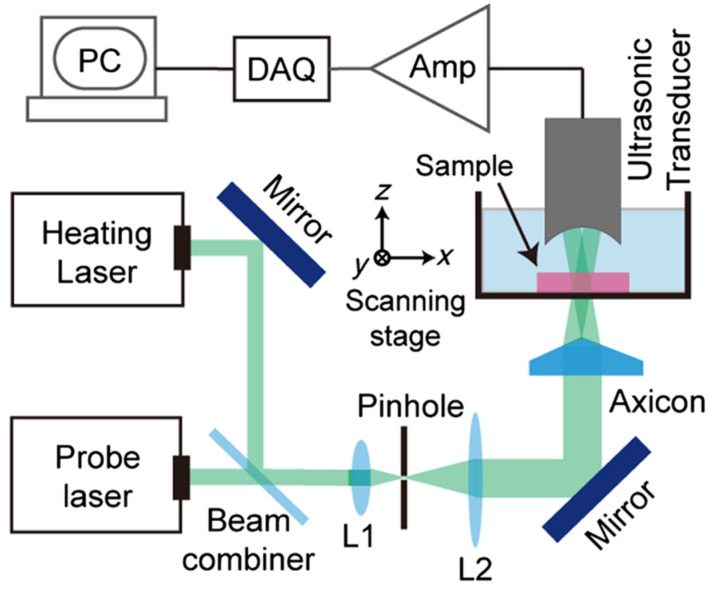
Schematic diagram of the Bessel beam photoacoustic microscopy system [28].

Acoustic and optical wavefront shaping techniques have emerged as powerful tools for enhancing the performance of imaging systems in recent decades. In PAI, wavefront shaping methods can be employed to improve spatial resolution, increase imaging depth, and enhance image quality. In 2011, Maslov et al. [74] introduced a second-generation OR-PAM system. This system incorporated a novel coaxial photoacoustic combiner and replaced the conventional right-angle prism with a rhomboid prism. This modification improved detection sensitivity by enhancing the conversion of transverse acoustic waves into longitudinal waves, which indirectly led to an improvement in spatial resolution [74,75]. The system achieved a lateral resolution of 2.6 μm, as determined using a USAF1951 resolution test chart. In 2023, Cao et al. utilized a custom-designed diffractive optical element (DOE) to generate a non-diffracting, needle-shaped beam, extending the depth of focus and reducing side lobes compared to conventional Gaussian beams. This approach led to an improvement in spatial resolution. Using a USAF1951 resolution test chart, they achieved a lateral resolution of 1.2 μm with this system [76]. Significantly, in 2020, Wang’s group employed an axicon lens to generate a Bessel beam, which is known for its non-diffracting properties and extended depth of focus. They achieved a lateral resolution of 6.1 μm using a 48 MHz ultrasonic transducer and a 0.25 NA objective lens to implement Bessel beam PAM [28]. Figure 20 presents a schematic diagram of the Bessel beam PAM system developed by Shi’s group.

While the implementation of wavefront shaping techniques in PAM can be technically demanding, integrating these methods with other advanced components, such as high-frequency transducers, high-NA objective lenses, and sophisticated scanning systems, holds significant promise for further enhancing spatial resolution and achieving breakthroughs in PAI capabilities. Currently, the improvement from waveform shaping may be limited due to its complex structure, potentially hindering its widespread clinical adoption.

#### 3.2.5. Nonlinear Effects

**Figure 21:**
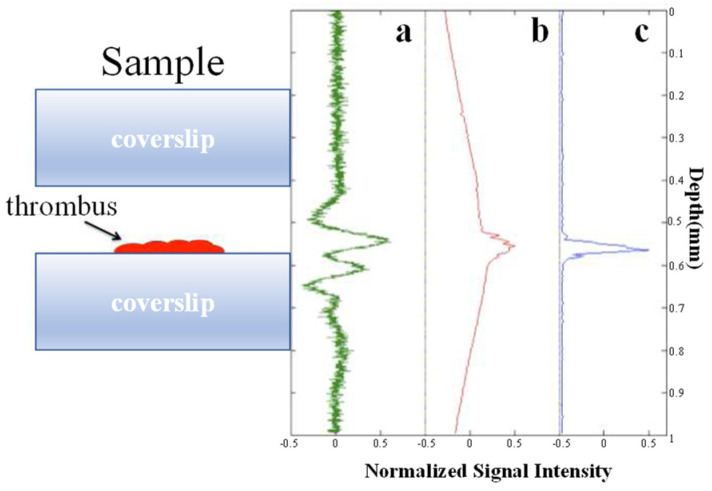
Axial scans of a blood clot sample [77]. Left panel: schematic representation of the sample. (**a**) Photoacoustic microscopy A-scan, with a nominal axial resolution of 300 μm. (**b**) Integrated photoacoustic signal, analogous to single-photon fluorescence microscopy. (**c**) TAUM axial scan, analogous to multiphoton microscopy, with a nominal axial resolution of 7 μm.

Nonlinear effects in photoacoustic imaging refer to nonlinear phenomena occurring during the interaction of light with matter, manifested as a nonlinear conversion of light energy by the medium, often achieved through pre-excitation of the medium. In 2010, Ryan L. Shelton and Brian E. Applegate utilized the transient absorption effect as a method to improve resolution in photoacoustic imaging, termed transient absorption ultrasonic microscopy (TAUM) [77]. Figure 21 illustrates the resolution improvement achieved through axial scanning in this microscopy technique. The transient absorption effect is a type of multiphoton absorption effect, referring to the phenomenon where a molecule undergoes an energy level transition upon absorbing two or more photons [78]. Its specific implementation involves using two beams of light or a modulated laser beam, serving as the pump and probe beams, respectively. First, the pump beam excites the sample, promoting it to an excited state and increasing its absorption, thereby enhancing the photoacoustic effect. The probe beam is primarily used for photoacoustic imaging. This multiphoton absorption process enables the signal intensity to be proportional to the square of the laser intensity, thereby improving axial resolution. Using the TAUM technique, the authors observed isolated hamster capillaries, achieving an axial resolution of 6 μm and a theoretical lateral resolution of 0.3 μm. In 2013, the research team further improved their imaging technique and observed fixed red blood cells and in vitro microvessels, achieving an axial resolution of 1.5 μm [79,80].

**Figure 22:**
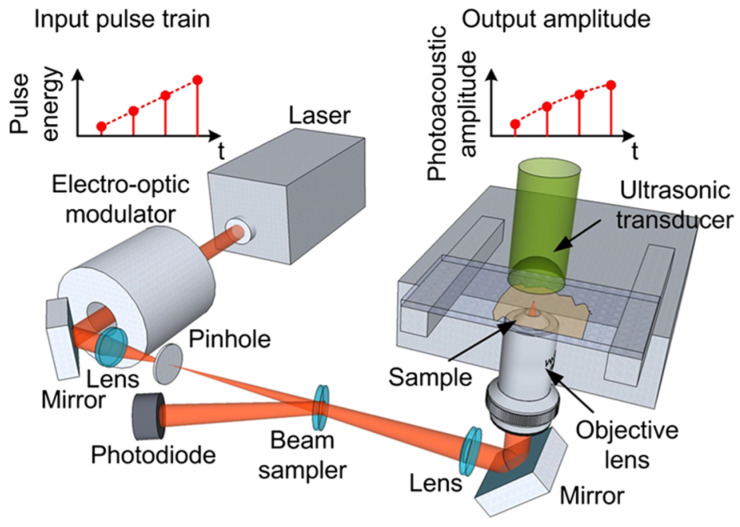
Schematic diagram of the photoacoustic nanomicroscopy system [81].

In 2014, Danielli et al. introduced a PAM system based on multi-pulse laser excitation. This system is capable of emitting a train of laser pulses with varying energies, either continuously or at defined time intervals. This multi-pulse illumination scheme exploits the thermal saturation effect, in which the absorption of optical energy by the sample decreases with increasing pulse energy or repetition rate, to generate nonlinear PA signals. By fitting a nonlinear polynomial function to the acquired series of PA signals and utilizing the higher-order terms of the polynomial, the spatial resolution of the PA images can be enhanced. While this technique demonstrated a lateral resolution of 88 nm for imaging isolated cells, it also faces challenges, including system complexity, high cost, and limited imaging depth [81]. Figure 22 presents a schematic diagram of the photoacoustic nanomicroscopy system developed by Danielli et al. [81].

In 2019, Shi et al. introduced a novel PAM system based on the Grüneisen relaxation effect. The Grüneisen parameter (Γ) is a thermodynamic property that relates the change in volume of a material to the change in its temperature [82]. The Grüneisen relaxation effect describes the transient change in Γ within a material upon rapid heating, typically induced by a pulsed laser. This change occurs on a timescale shorter than the thermal relaxation time of the material, influencing the generation of the PA signal. This PAM system employed infrared (IR) light for excitation and UV light for probing, potentially enabling enhanced spatial resolution due to the shorter wavelength of UV light. In 2020, the same group developed a PAM system that combined Bessel beam illumination with the Grüneisen relaxation effect. They leveraged the non-diffracting properties of Bessel beams and the nonlinear dependence of the PA signal on excitation intensity, arising from the Grüneisen relaxation effect, to simultaneously improve axial resolution and mitigate side lobes inherent to Bessel beam illumination [28]. Using a 0.25 NA objective lens and a 48 MHz focused ultrasonic transducer, they achieved a lateral resolution of 6.1 μm and an axial resolution of 30 μm with their Bessel beam-based Grüneisen relaxation PAM system. This novel approach represents a promising direction for further development of high-resolution PAM systems. Furthermore, leveraging the Grüneisen relaxation effect potentially enables the axial resolution in PAI to be decoupled from the bandwidth limitations of the ultrasonic transducer, instead becoming more dependent on the optical properties of the sample and the excitation pulse duration. This opens up new avenues for improving the axial resolution of PAI systems. In clinical applications, optimizing photoacoustic microscopy using nonlinear effects may be limited by system complexity and immature operational procedures, and its theoretical basis may require further validation through practical cases.

#### 3.2.6. Deep Learning Algorithms

**Figure 23:**
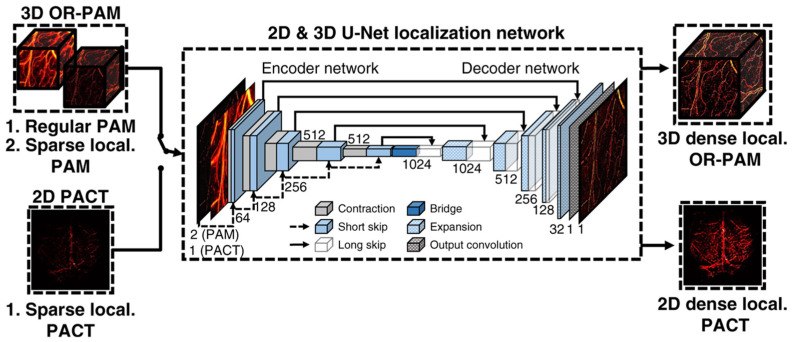
Visual representation of the 2D and 3D U-Net generative network architectures employed by Jongbeom Kim [29].

Analogous to AR-PAM, iterative and deep learning-based algorithms are increasingly being utilized in OR-PAM to enhance spatial resolution and image quality. In 2020, Pleitez et al. demonstrated the use of bicubic interpolation and iterative Wiener deconvolution to improve image quality in OR-PAM. They performed slice-based imaging of the mouse pancreas using mid-infrared wavelengths, achieving a lateral resolution of 5.3 μm [83]. In 2022, Kim et al. applied deep neural network (DNN) and GAN models to super-resolution techniques in OR-PAM, enabling the generation of high-resolution images from a limited number of sparsely sampled PA images [29]. Figure 23 depicts the architecture of the U-Net generative network employed in their study. In 2024, Loc et al. proposed DiffPAM, an algorithm based on a pre-trained unsupervised diffusion model, for high-resolution image reconstruction from undersampled PAM data. The DiffPAM algorithm mitigates the large training dataset requirements typically associated with GAN-based approaches [84]. In summary, deep learning techniques are rapidly being adopted across various fields, including PAI, where they are driving significant advancements in image processing, resolution enhancement, and system performance. Similar to acoustic-resolution photoacoustic microscopy, deep learning can also be integrated with existing technologies and effectively applied in clinical diagnosis.

Table 5 presents the clinical usability, advantages, and disadvantages of optical-resolution photoacoustic microscopy.

## 4. Conclusions

Photoacoustic imaging (PAI), a hybrid imaging modality that leverages the advantages of both optical and acoustic waves, has matured significantly over the past few decades. Researchers are increasingly integrating techniques from other disciplines, including optics, acoustics, materials science, and computer science, with innovative approaches to further advance PAI capabilities. Current research in PAI is focused on integrating cutting-edge technologies, such as deep learning, and on achieving miniaturization, high-speed imaging, enhanced spatial resolution, and multi-modal capabilities. Despite the significant progress achieved in PAM, several challenges remain to be addressed for its widespread adoption in clinical and preclinical settings. These challenges include (1) improving imaging depth while maintaining high spatial resolution; (2) increasing imaging speed, particularly by overcoming the limitations of mechanical scanning mechanisms; (3) developing novel imaging systems based on nonlinear effects to decouple axial resolution from the bandwidth limitations of ultrasonic transducers; and (4) reducing the cost and complexity of PAM systems. (5) Miniaturization and portability have always been the development trends of modern medical devices, and photoacoustic imaging will also achieve device miniaturization in the future with the help of advanced optical and acoustic components and optimized structural configurations. (6) Due to its combined optical and acoustic characteristics, photoacoustic imaging will be deeply integrated with other imaging techniques such as ultrasound imaging, OCT (optical coherence tomography), and fluorescence imaging in the future, forming multimodal and multifunctional detection systems. (7) At the clinical application level, relevant regulations and guidelines are not yet fully developed, and researchers are still required to provide a large amount of laboratory and clinical data as support.

Overcoming these obstacles will be critical for translating the promising capabilities of PAI from laboratory research into practical applications in biomedicine and other fields. PAI has already shown its potential for numerous biomedical applications, including measuring red blood cell oxygenation, monitoring red blood cell metabolism, and visualizing DNA and RNA structures. As PAI technology continues to advance, it is poised to become an indispensable tool for biomedical imaging and diagnostics, offering unique capabilities for visualizing biological structures, monitoring physiological processes, and detecting disease at various scales.

## Figures and Tables

**Table 1:** Methods for improving resolution in acoustic-resolution photoacoustics.

Methods	Description	References
Transducer optimization	Utilizing transducers with high numerical aperture (NA) and high central frequency, or designing novel transducers specifically tailored for photoacoustic microscopy.	[21,31,32,33,34]
Synthetic aperture focusing technique (SAFT)	Utilizing a single-element transducer to scan and simulate a transducer with a larger numerical aperture, combined with signal processing to achieve resolution enhancement.	[35,36,37,38]
Neural network algorithms	Utilizing neural network algorithms to process photoacoustic images and thereby enhance resolution.	[39]

**Table 2:** The clinical usability and the associated advantages and disadvantages of acoustic-resolution photoacoustic microscopy.

Methods	Resolution Improvement	Cost	Universality	Clinical Usability
Improving transducers [21,31,32,33,34]	Very high. The improvement effect is quite significant.	High. The processing difficulty of high-precision and high-numerical-aperture optical or acoustic devices is relatively high.	High. Through reasonable design, it is applicable to any scenario.	High. Through reasonable design, it is easy to apply in clinical practice.
Synthetic aperture focusing technology (SAFT) [35,36,37,38]	High. The improvement effect is average.	Higher. Requires precise scanning equipment and algorithms.	High. Can be applied to most photoacoustic microscopy imaging environments.	Generally. The scanning speed is slow and difficult to apply in clinical practice.
Neural network algorithm [39]	Generally.	Generally. Requires a large amount of data for training.	High. Can be applied to various imaging scenarios.	High. It can be well applied in clinical diagnosis.

**Table 3:** Methods for enhancing resolution in optical-resolution photoacoustic microscopy.

Methods	Description	References
Enhancement of the optical objective or transducer	Utilizing high-numerical-aperture (NA) optical objectives and high-center-frequency transducers, or employing novel optical or acoustic components specifically designed for photoacoustic microscopy.	[11,20,23,58,59,60,61,62,63]
Enhancing scanning devices or scanning methods	Primarily represented by real-time 3D profiling scanning techniques, this involves the application of precise scanning technologies or the utilization of improved scanning devices.	[27,64,65]
Exploiting specific light absorption	Based on the characteristics of the object under investigation, utilizing lasers with specific wavelengths, or employing contrast agents to enhance the photoacoustic signal based on the properties of the illuminating laser.	[66,67,68,69,70,71,72,73]
Acoustic or optical wavefront shaping	Utilizing specialized optical or acoustic components to shape optical or acoustic waves, respectively, for achieving high resolution.	[28,74,75,76]
Nonlinear effects	Utilizing nonlinear effects such as two-photon absorption and Grueneisen relaxation, along with signal processing methods like polynomial fitting, to enhance resolution.	[28,77,78,79,80,81,82]
Deep learning algorithms	Employing deep learning algorithms to process photoacoustic images, thereby enhancing resolution.	[29,83,84]

**Table 4:** Summary of common photoacoustic contrast agents [66].

Category	Material Type	Properties and Description
Inorganic photothermal conversion agents	Gold nanoparticles	High photothermal conversion efficiency, tunable surface plasmon resonance (SPR) peak.
	Silver nanoparticles	High optical absorption coefficient, but prone to oxidation and stability challenges.
	Carbon-based materials (e.g., graphene)	Exceptional photothermal stability, structural integrity, and electrical conductivity, rendering them well suited for photoacoustic imaging of deep tissues.
	2D materials (e.g., black phosphorus)	Possessing a unique two-dimensional structure, it can provide high-contrast photoacoustic imaging.
Organic photothermal conversion agents	Small molecules with near-infrared (NIR) responsiveness	Chemical modification allows for the adjustment of the absorption spectrum.
	Semiconducting polymeric nanoparticles	Photothermal properties can be optimized through the design of molecular structures.
Photoacoustic imaging probes	Organic chromophores	With distinct absorption peaks, they serve to amplify photoacoustic signals at specific wavelengths.
	Quantum dots	High photostability and tunable emission wavelengths render them suitable for multimodal imaging applications.

**Table 5:** The clinical usability, advantages, and disadvantages of optical-resolution photoacoustic microscopy.

Methods	Resolution Improvement	Cost	Universality	Clinical Usability
Improving optical objectives or transducers [11,20,23,58,59,60,61,62,63]	Very high. The improvement effect is quite significant.	High. The processing difficulty of high-precision and high-numerical-aperture optical or acoustic devices is relatively high.	High. Through reasonable design, it is applicable to any scenario.	High. Through reasonable design, it is easy to apply in clinical practice.
Improving scanning devices or scanning methods [27,64,65]	High.	High. Precise scanning machinery or motors are required.	Limited. It is difficult to apply to scenarios that require high scanning speed.	Generally. It has guiding significance.
Utilizing specific light absorption [66,67,68,69,70,71,72,73]	High. The ultraviolet band has a significant effect.	Generally. The price of enhancers varies.	Limited. The application scenario is limited by the use of enhancers.	Good. It can be conveniently applied to clinical practice in specific scenarios.
Shaping sound waves or light waves [28,74,75,76]	Poor.	Generally. Special equipment may need to be customized.	Limited. Special design is required for the experiment.	Poor. The structure is relatively complex.
Nonlinear effects [28,77,78,79,80,81,82]	Good. There are cases with significant effects.	High. Customized lasers and special structural designs are required.	Limited. Restricted by specific substances.	Poor. Poor energy controllability, not theoretically verified.
Deep learning algorithms [29,83,84]	Generally.	Generally. Requires a large amount of data for training.	High. Can be applied to various imaging scenarios.	High. It can be well applied in clinical diagnosis.
